# AARP PUBLIC POLICY INSTITUTE PRESENTS THE LONG-TERM SERVICES AND SUPPORTS STATE SCORECARD, 2023

**DOI:** 10.1093/geroni/igad104.0588

**Published:** 2023-12-21

**Authors:** Susan Reinhard, Susan Reinhard

## Abstract

This symposium will present and explore in-depth the 2023 Long-Term Services and Supports (LTSS) State Scorecard. With funding from The SCAN Foundation, The Commonwealth Fund, and The John A. Hartford Foundation, this Scorecard is the fifth edition from the AARP Public Policy Institute. The Scorecard ranks states across five key dimensions that comprise a framework for high-performing LTSS system performance: affordability and access, choice of setting and provider, quality and safety, support for family caregivers, and community and integration. In this symposium, speakers will discuss the Scorecard’s history, its most recent findings, and the implications of the COVID-19 pandemic for state LTSS system performance. In addition, the Scorecard explores LTSS equity, and we will discuss how policymakers, practitioners, the private sector, and other actors can further realize equitable systems of supports.

